# Down-regulation of SerpinB2 is associated with gefitinib resistance in non-small cell lung cancer and enhances invadopodia-like structure protrusions

**DOI:** 10.1038/srep32258

**Published:** 2016-08-25

**Authors:** Song Yi Bae, Hyen Joo Park, Ji-Young Hong, Hye-Jung Lee, Sang Kook Lee

**Affiliations:** 1 College of Pharmacy, Natural Products Research Institute, Seoul National University, Seoul 151-742, Korea

## Abstract

The failure of targeted therapy due to the resistance to epidermal growth factor receptor tyrosine kinase inhibitors (EGFR-TKIs), such as gefitinib, is considered a major problem in the treatment of non-small cell lung cancer (NSCLC) patients. SerpinB2, a component of the urokinase plasminogen activator (uPA) system, has been recognized as a biomarker for the progression and metastasis of lung cancer. Nevertheless, the relationship between SerpinB2 and EGFR-TKI resistance has not been elucidated. Here, we report that SerpinB2 is down-regulated in gefitinib-resistant (H292-Gef) cells compared to gefitinib-sensitive (H292) cells. The low SerpinB2 levels in H292-Gef cells were also associated with an enhancement in invasiveness and increase in the length of invadopodia-like structures in the cells. The effect on invasiveness and gefitinib sensitivity was confirmed by knockdown and overexpression of SerpinB2. In addition, the possibility to overcome the resistance through the up-regulation of SerpinB2 was supported by employing an antitumor agent yuanhuadine (YD). Treatment with YD effectively elevated SerpinB2 levels and suppressed invasive properties in H292-Gef cells. Collectively, these findings demonstrate the prospective role of SerpinB2 as a novel biomarker for acquired gefitinib resistance and a potential target for NSCLC treatment.

Non-small cell lung cancer (NSCLC) is one of the leading causes of cancer-related death worldwide. Despite the development of novel chemotherapeutic agents and regimens for lung cancer treatment, acquired and inborn drug resistance, including epidermal growth factor receptor tyrosine kinase inhibitor (EGFR-TKI) resistance, have been major barriers for chemotherapy. Therefore, studies have focused on identifying potential prognostic and drug resistant markers, such as EGFR, KRAS, and AXL, in lung cancers^1^,^2^,^3^. Over the past 20 years, the levels of SerpinB2 expression in NSCLC has been proposed to be a potential biomarker for cancer progression^4^,^5^,^6^. Low SerpinB2 levels are correlated with high metastatic characteristics in human lung cancer cells, lymph node metastasis and poor prognosis in primary lung cancer.

SerpinB2 is a member of the Clade B subgroup of the serine protease inhibitor (serpin) superfamily and is also known as plasminogen activator inhibitor type 2 (PAI-2) due to its inhibitory activity against serine protease plasminogen activators^7^. SerpinB2 is one of the primary components of the urokinase plasminogen activator (uPA) system, which includes uPA, the membrane-linked receptor uPAR and SerpinE1 (also known as PAI-1). The uPA system is involved in the regulation of extracellular matrix (ECM) degradation. Active uPAR-bound uPA converts inactive plasminogen to plasmin, which directly degrades ECM molecules, releases latent growth factors, and indirectly breaks down ECM molecules through the activation of pro-matrix metalloproteinases (pro-MMPs)^8^,^9^. The role of SerpinB2 and SerpinE1 in the uPA system is to inhibit uPA through the formation of non-reversible covalent complexes with uPA. These complexes then interact with low-density lipoprotein receptor-related proteins (LRP) to promote endocytosis, followed by degradation^10^,^11^. Additionally, SerpinE1 directly interacts with the ECM component vitronectin, LRP and the very-low-density lipoprotein receptor (VLDLR), which results in increased cell adhesion, migration and proliferation^12^,^13^. Unlike SerpinE1, SerpinB2 does not participate in these interactions and cannot induce these effects^14^.

Extensive studies have suggested that the up-regulation of the uPA system enhances tumor cell proliferation, invasion, metastasis and tumor angiogenesis^15^,^16^. Accordingly, clinical results have identified high levels of uPA, uPAR and SerpinE1 to be markers of poor prognosis and outcomes in various cancer types^17^. In contrast, decreased SerpinB2 levels have been correlated with unfavourable outcomes in breast^18^, head and neck^19^, gastric^20^ and liver^21^ cancers. Moreover, a recent study reported that the down-regulation of SerpinB2 is associated with an acquired resistance to cisplatin in head and neck squamous cell cancer^22^.

The occurrence of metastasis is one of the major causes in cancer progression and poor drug-response. During metastasis, cancer cells disseminate from the primary site to secondary site in distant organs through cellular migration and invasion. Cancer cells gain enhanced migratory and invasive properties by remodeling the actin cytoskeleton and by forming invasive structures such as lamellipodia, filopodia, invadopodia and podosomes^23^,^24^. In general, lamellipodia and filopodia play a role in horizontal movement within two-dimensional culture; however, invadopodia and podosomes are required to move into or through a three-dimensional matrix, which is similar to the *in vivo* situation. Invadopodia strongly degrade ECM for up to one hour, whereas podosomes are less able to degrade the ECM and have a short lifespan of a few minutes. Therefore, the suppression of the migratory and invasive characteristics mediated by drug-resistant cancer cells could be an attractive target for overcoming resistance.

Although several reports have identified SerpinB2 as an important marker for lung cancer progression and metastasis, the relationship between SerpinB2 and EGFR-TKI resistance has not been clearly elucidated. Here, we demonstrated for the first time that SerpinB2 levels are down-regulated in NSCLC cells with acquired resistance to gefitinib, an EGFR-TKI. The down-regulation of SerpinB2 increased the invasiveness of gefitinib-resistant cells by lengthening invadopodia-like structures, the protrusions observed around the whole cell surface^25^,^26^. Yuanhuadine (YD)^27^, an anti-tumor agent, was used as a SerpinB2-inducing agent to confirm the role of SerpinB2 in EGFR-TKI resistance. These findings suggest that SerpinB2 may be a possible marker for gefitinib resistance and a target for the treatment of gefitinib-resistant NSCLC.

## Results

### SerpinB2 expression is decreased in gefitinib-resistant H292 (H292-Gef) cells

**Figure 1:**
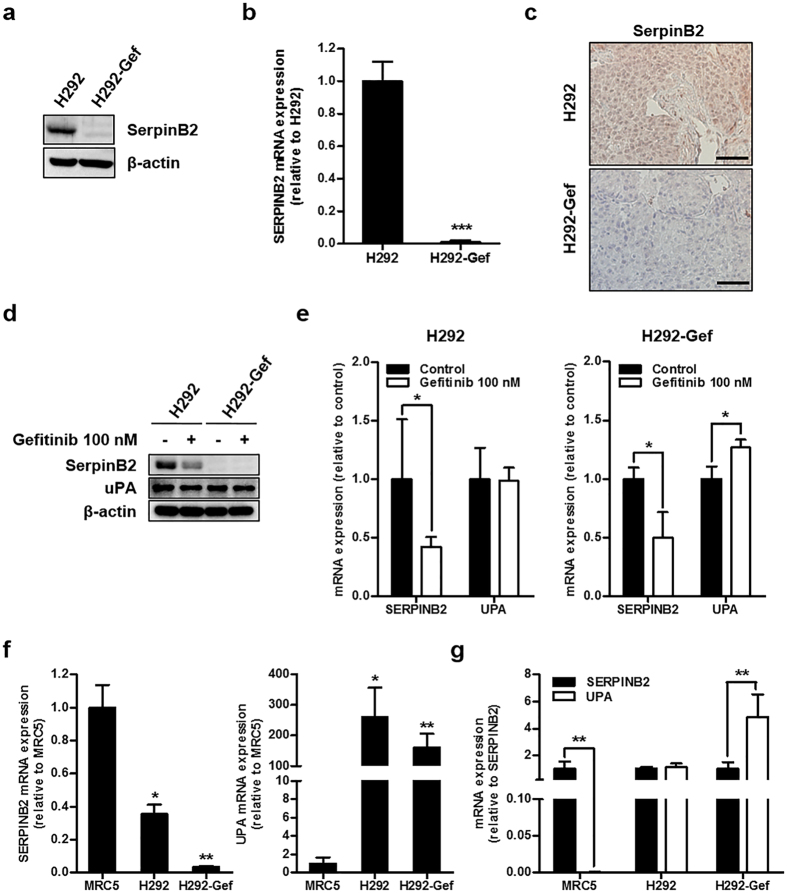
Down-regulation of SerpinB2 in gefitinib-resistant lung cancer cells. **(a)** Cells were lysed, and SerpinB2 levels were analysed by western blot with an antibody against SerpinB2 and using β-actin as a loading control. **(b)** The mRNA levels of SERPINB2 were examined using real-time PCR. The data are presented as the mean fold changes ± SD relative to the H292 control. **(c)** Immunohistochemical analysis of SerpinB2 was performed using an anti-SerpinB2 antibody in tumor tissue sections obtained from a previous study. Scale bar, 50 μm. **(d,e)** Cells were treated with 100 nM gefitinib for 24 h and then SerpinB2 and uPA protein (**d**) and mRNA (**e**) levels were detected by western blot and real-time PCR, respectively. The data are presented as the mean fold changes ± SD relative to the vehicle-treated control. **(f)** SERPINB2 and UPA mRNA expression was examined using real-time PCR. The data are presented as the mean fold changes ± SD relative to the MRC-5 control. **(g)** SERPINB2 and UPA mRNA levels are presented as the mean fold changes ± SD relative to SERPINB2 expression of indicated cells. β-actin mRNA levels were used for normalization of real-time PCR data. *P < 0.05, **P < 0.01, ***P < 0.005.

In our previous study, the gefitinib-resistant NSCLC cell line H292-Gef was established from a gefitinib-sensitive cell line, H292^28^. A cDNA microarray was performed to identify the genes that were differentially expressed between these two cell lines, and using the standard 2-fold change in expression as our threshold criterion, gene expression analysis revealed several significant changes between the two cell lines (Table 1). From this analysis, we found a significant down-regulation in SERPINB2 expression in H292-Gef cells compared to the H292 cells. The differential protein and gene expressions of SerpinB2 between the two cell lines were reconfirmed *in vitro*; SerpinB2 protein was barely detectable in H292-Gef cells (Fig. 1a). Consistent with this protein expression, the mRNA expression of SERPINB2 was decreased approximately 100-fold compared to H292 cells (Fig. 1b). Analysis of tumor tissues obtained from the previous *in vivo* study^28^ also demonstrated the down-regulated protein and gene expressions of SerpinB2 in H292-Gef cells-implanted compared to H292 cells-implanted xenograft tumors (Fig. 1c and Supplementary Fig. S1). In addition, the expression of SerpinB2 was also found to be down-regulated in an acquired gefitinib-resistant NSCLC cell line, H1993-Gef cell (Supplementary Fig. S2).

To evaluate the effect of gefitinib on SerpinB2 expression, cells were treated with 100 nM gefitinib for 24 h. Alterations in SerpinB2 expression in H292-Gef cells were not observed due to its low basal level of protein expression; however, decreased expression was detected in H292 cells following gefitinib treatment (Fig. 1d). Despite the change of SerpinB2 levels in H292 cells, the anti-proliferative and anti-invasive activities of gefitinib were maintained (Supplementary Fig. S3). The expression of SerpinB2 is highly related to its target, uPA, in the prognosis of cancer patients^14^. However, there were no changes in uPA protein levels in either cell line following gefitinib treatment. In addition, we determined the mRNA expression levels of SERPINB2 and UPA (also known as PLAU). The mRNA fold-differences were slightly different from the changes in protein expression: SERPINB2 expression was down-regulated by gefitinib in both H292 and H292-Gef cells, and UPA expression was up-regulated only in H292-Gef cells (Fig. 1e).

The expression levels of these two genes were further compared to those in the normal lung cell line, MRC5. SERPINB2 was decreased while UPA was increased in cancer cells compared to MRC5 cells (Fig. 1f). Compared to SERPINB2 expression, UPA levels were relatively lower in MRC5 cells and higher in H292-Gef cells; therefore, the ratio between two genes was different in normal and cancer cells (Fig. 1g). Taken together, continuous treatment of the parental cell line, H292, with gefitinib may have resulted in the down-regulation of SerpinB2 expression and a change in the ratio of expression between SERPINB2 and UPA in H292-Gef cells compared to normal lung cells.

### The invasiveness and length of invadopodia-like structures are increased in H292-Gef cells

**Figure 2:**
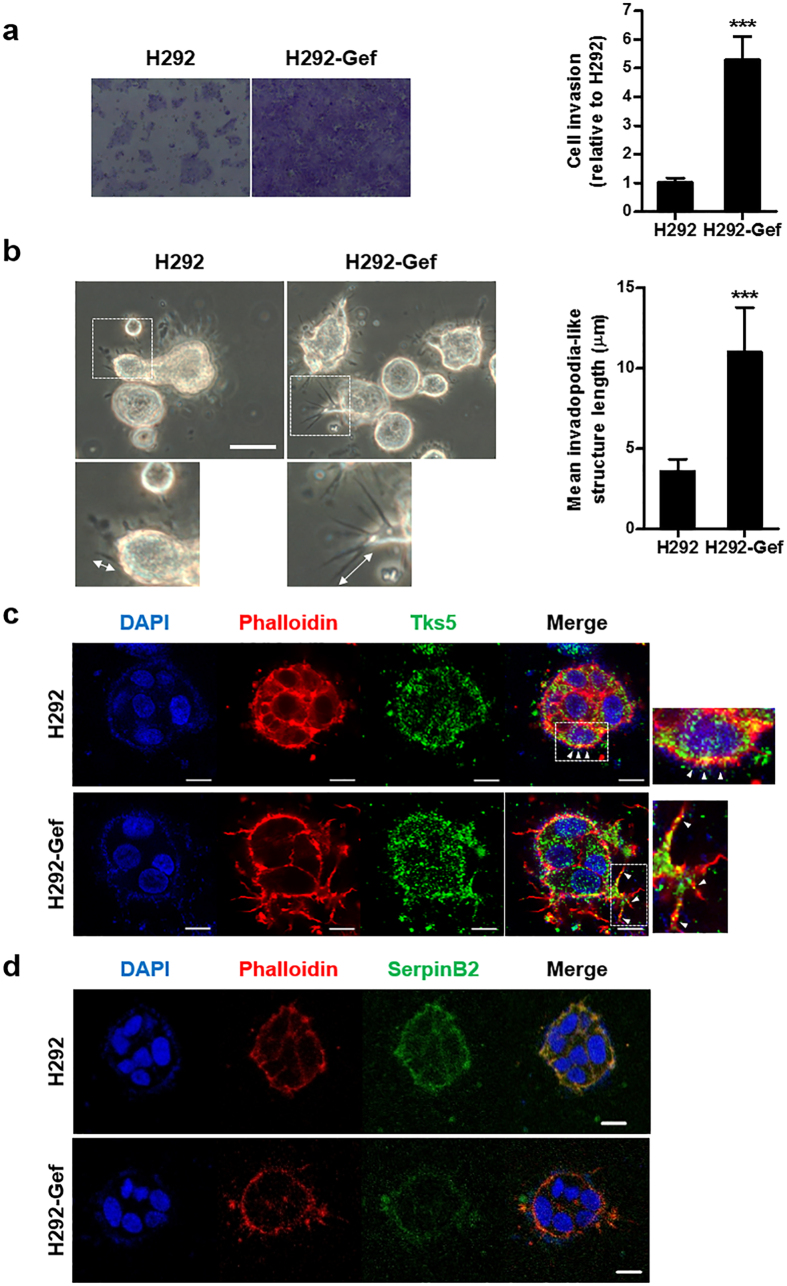
Differences in invasive ability and length of invadopodia-like structures in H292 and H292-Gef cells. **(a)** The invasion assay was performed as described in the Methods section. Cells suspended in serum-free medium were added to the upper chambers of Matrigel-coated transwell inserts. The lower chambers contained medium supplemented with 10% FBS. Following a 24-h incubation period, the invaded cells on the lower surface of the membrane were fixed, stained, counted and photographed. Magnification, x100. The data are presented as the mean ± SD. **(b)** Cells were embedded into 3D collagen I gels and incubated for 2 days. On Day 2, cells were visualized by light microscopy and length of invadopodia-like structures was measured. The data are presented as the mean ± SD. Scale bar, 25 μm. **(c,d)** 3D-cultured cells were stained with anti-phalloidin, anti-Tks5, anti-SerpinB2 and DAPI for immunocytochemical analysis. Scale bar, 10 μm. Arrows in insets of (**c**) indicate invadopodia-like structures. *P < 0.05, **P < 0.01, ***P < 0.005.

Because of its role in the uPA system, the down-regulation of SerpinB2 could result in an increase in the invasiveness of H292-Gef cells. Indeed, we observed an approximately 5-fold increase in the invasive ability of H292-Gef cells compared to the H292 cells (Fig. 2a). The overexpression of SerpinB2 in mouse melanoma cells has been reported to reduce the length of invadopodia-like structures while not affecting their initial formation, and this event consequently reduced the migration and invasion of cells^25^. Based on these findings, we evaluated the lengths of invadopodia-like structures in the H292 and H292-Gef cells. The cells were cultured in 3D collagen gels and were examined under a phase-contrast microscope, and we observed that the invadopodia-like structures were longer in H292-Gef cells compared to H292 cells (Fig. 2b). Invadopodia are highly enriched with actin filaments (F-actin) and actin assembly machinery, including the Arp2/3 actin nucleation complex, neural-Wiskott Aldrich Syndrome protein (N-WASP) and cortactin^29^. Accordingly, we performed immunocytochemical staining of the 3D-cultured cells using fluorescent-conjugated phalloidin for F-actin staining. We also detected the protrusions with the adaptor protein and Src substrate Tks5, a specific biomarker for invadopodia^30^. We confirmed that the protrusion of invadopodia-like structures was increased in H292-Gef cells (Fig. 2c,d). Moreover, SerpinB2 expression levels were decreased in H292-Gef cells (Fig. 2d). Collectively, SerpinB2 is down-regulated in H292-Gef cells, and this may result in an increase in their invasive ability through the lengthening of invadopodia-like structures.

### SerpinB2 contributes to the elongation of invadopodia-like structures in H292-Gef cells

**Figure 3:**
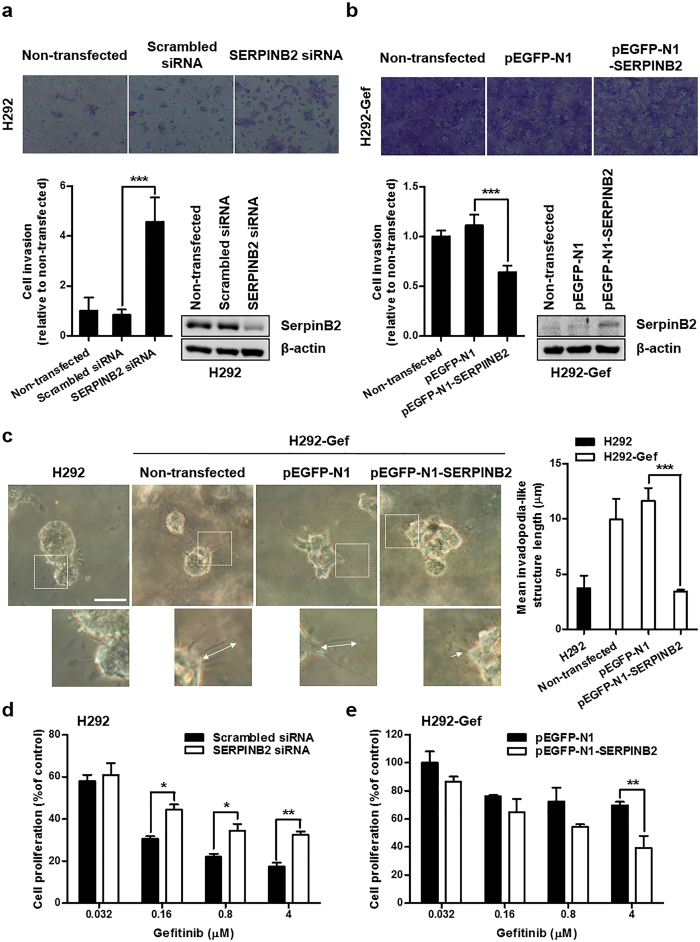
SerpinB2-dependent changes in invasive characteristics and gefitinib sensitivity in H292 and H292-Gef cells. **(a,b)** H292 (**a**) and H292-Gef (**b**) cells were not transfected or transfected with indicated siRNAs or plasmids for 24 h, and altered gene expression was confirmed by western blot using a SerpinB2 antibody. The cell invasion assay was performed with transfected cells as described in the Methods section. Magnification, x100. The data are presented as the mean ± SD. **(c)** Cells were embedded into 3D collagen I gels and incubated for 2 days. On Day 2, cells were visualized by light microscopy and length of invadopodia-like structures was measured. The data are presented as the mean ± SD. Scale bar, 25 μm. **(d,e)** H292 (**d**) and H292-Gef (**e**) cells were transfected with indicated siRNAs or plasmids for 24 h. The transfected cells were plated in 96-well culture plates for 24 h and then treated with gefitinib for 48 h. The data are presented as the mean ± SD. *P < 0.05, **P < 0.01, ***P < 0.005.

To investigate whether decreased SerpinB2 expression is related to increased length of invadopodia-like structures and the subsequent invasiveness of H292-Gef cells, H292 and H292-Gef cells were transfected with a SERPINB2 small interfering RNA (siRNA) or a SERPINB2-expressing plasmid (pEGFP-N1-SERPINB2). The efficiency of the pEGFP-N1-SERPINB2 construct was verified prior to its use. pEGFP-N1-SERPINB2-transfected H292-Gef cells expressed GFP, and SERPINB2 mRNA expression was increased by more than 30-fold compared to cells transfected with pEGFP-N1, the empty vector used for cloning (Supplementary Fig. S4a,b). The protein level of SerpinB2 was also increased and was detected with a molecular weight of ~74 kDa due to GFP tagging (Supplementary Fig. S4c). When we evaluated the effect of SerpinB2 expression changes on the invasive ability of the two cell lines, the invasion of H292 cells transfected with SERPINB2 siRNA was increased more than 4-fold compared to scrambled siRNA-transfected H292 cells (Fig. 3a). In contrast, H292-Gef cells transfected with pEGFP-N1-SERPINB2 showed a decrease in invasiveness compared to pEGFP-N1-transfected H292-Gef cells (Fig. 3b). The transfection efficiencies of the siRNAs and plasmids were shown by changes in SerpinB2 protein levels (Fig. 3a,b, right panel). Furthermore, the overexpression of SerpinB2 in H292-Gef cells resulted in the shortening of invadopodia-like structures (Fig. 3c). In addition, the alteration of SerpinB2 expressions also affected the sensitivity of two cell lines to gefitinib; SERPINB2-knockdown decreased gefitinib sensitivity of H292 cells, while SERPINB2-overexpression increased that of H292-Gef cells (Fig. 3d,e). Collectively, the level of SerpinB2 expression is associated with the sensitivity of gefitinib and low SerpinB2 levels in H292-Gef cells result in an increase in the length of invadopodia-like structures.

### Up-regulation of SerpinB2 by an antitumor agent suppresses the invasiveness of H292-Gef cells

**Figure 4:**
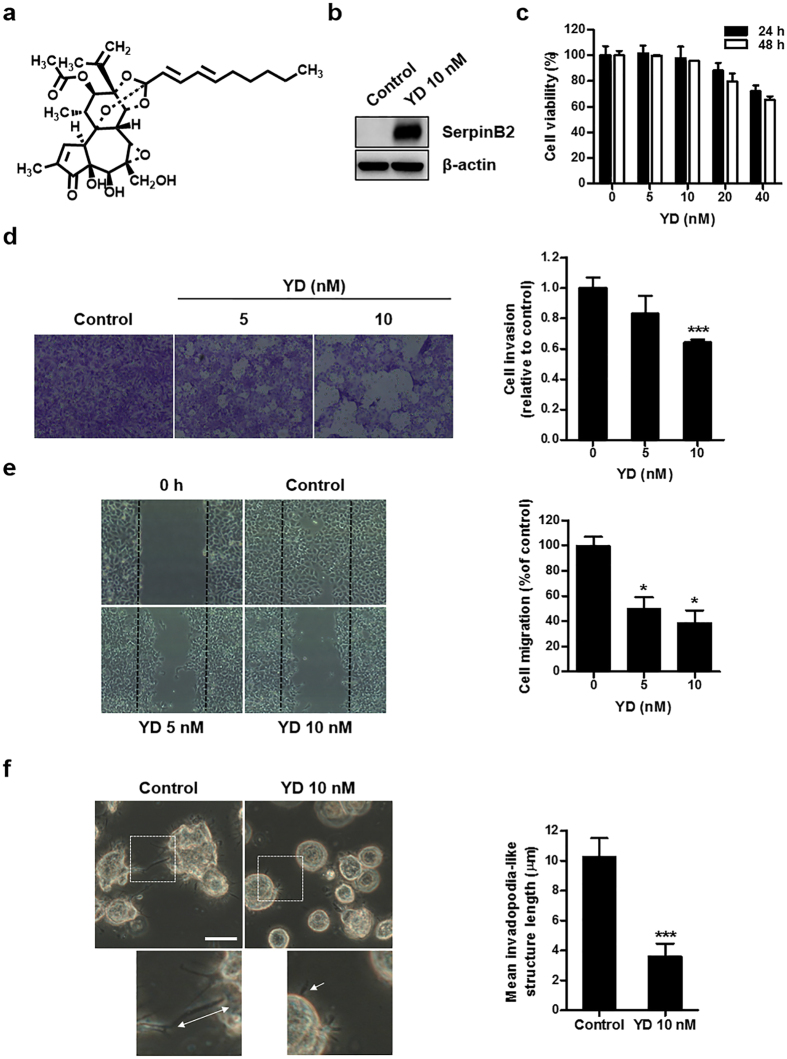
Effects of YD on the invasion and invadopodia-like structures in H292-Gef cells. **(a)** Structure of yuanhuadine (YD). **(b)** H292-Gef cells were treated with 10 nM YD for 24 h and then lysed and subjected to western blot using anti-SerpinB2 and β-actin as an internal standard. **(c)** H292-Gef cells were treated with indicated concentrations of YD for 24 and 48 h, and cell viability was then determined using the MTT assay. The data are presented as the mean ± SD. **(d)** H292-Gef cells were added to Matrigel-coated transwell inserts with YD and incubated for 48 h. The invaded cells were fixed, stained and counted as described in the Methods section. Magnification, x100. The data are presented as the mean ± SD. **(e)** After mechanistically generating scratches in the monolayer of H292-Gef cells, cells were treated with YD for 30 h, and wound closure was observed under a light microscope. The percentage of cell migration was calculated as described in the Methods section. The data are presented as the mean ± SD. Magnification, x40. **(f)** H292-Gef cells embedded in 3D collagen I gels were treated with 10 nM YD for 24 h. Cells were visualized by light microscopy and length of invadopodia-like structures was measured. The data are presented as the mean ± SD. Scale bar, 25 μm. *P < 0.05, **P < 0.01, ***P < 0.005.

To further explore the potential of targeting the down-regulated SerpinB2 expression to overcome gefitinib resistance, we applied YD (Fig. 4a), an antitumor agent that effectively inhibits the proliferation of NSCLC cells and exhibits synergistic effect when combined with gefitinib^27^,^28^,^31^. Treatment with 10 nM YD for 24 h dramatically increased SerpinB2 expression in H292-Gef cells (Fig. 4b). Based on this finding, we next examined the effect of YD on the invasive ability of H292-Gef cells. We used the MTT assay to determine the cytotoxicity of 24- and 48-h treatments of YD. Cell viability remained greater than 90% following 5- and 10-nM YD treatment (Fig. 4c). YD (5 and 10 nM) effectively suppressed the invasive and migratory abilities of H292-Gef cells (Fig. 4d,e). Consistent with the results shown in Fig. 3c, treatment with 10 nM YD reduced the length of invadopodia-like structures (Fig. 4f). Collectively, YD induced an up-regulation of SerpinB2, suppressing the invasive properties of H292-Gef cells and shortening invadopodia-like structures.

### YD regulates the expression of SerpinB2 and uPA in H292-Gef cells

**Figure 5:**
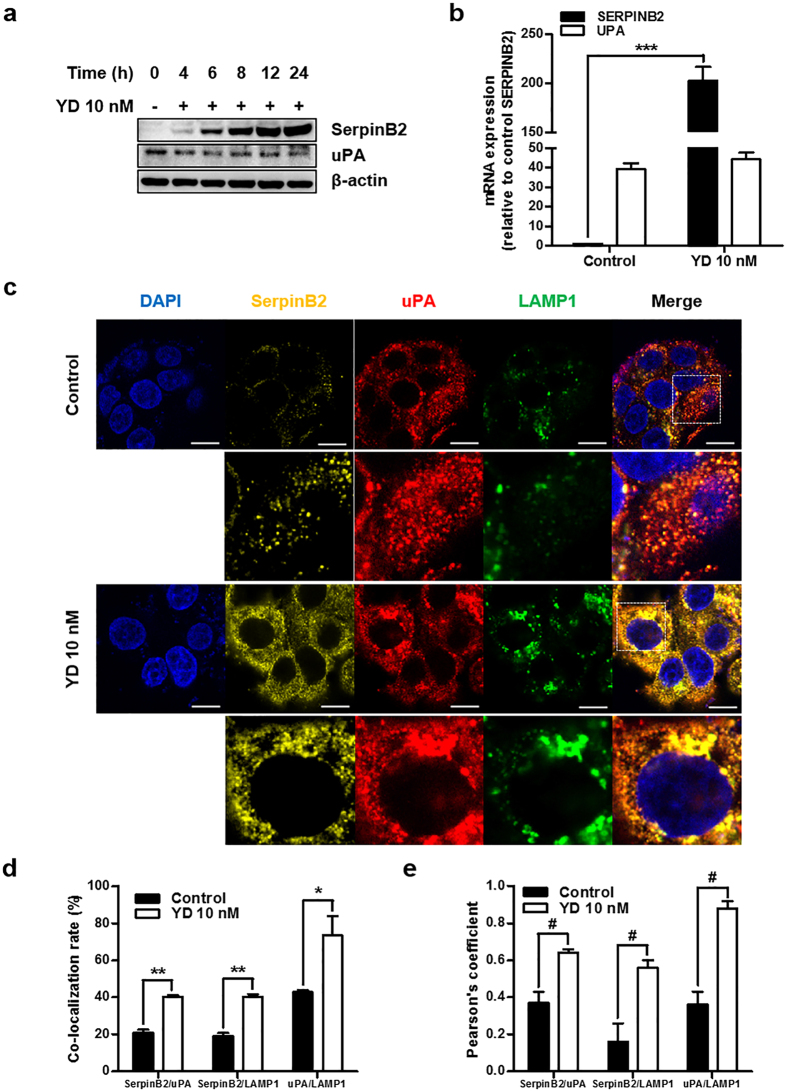
Effects of YD on uPA expression in H292-Gef cells. **(a**) H292-Gef cells were treated with 10 nM YD for the indicated times. The protein levels of SerpinB2 and uPA were examined by western blot analysis. **(b)** The mRNA levels of SERPINB2 and UPA were determined in H292-Gef cells following 24 h treatment with 10 nM YD. β-actin was used for normalization. The data are presented as the mean fold changes ± SD relative to the SERPINB2 of control. **(c)** Following treatment with 10 nM YD for 6 h, H292-Gef cells were fixed, blocked and stained with anti-SerpinB2, anti-uPA, anti-LAMP1 and DAPI. Images were taken using a confocal laser microscope. Scale bar, 10 μm. Boxed regions in merged images are enlarged and shown below each markers. **(d,e)** The co-localization of indicated markers were quantitatively analysed using the LAS AF Image software (Leica), and expressed as the percentages of co-localization rate (d) and Pearson’s coefficients (**e**). The data are presented as the mean ± SD. *P < 0.05, **P < 0.01, ***P < 0.005. Comparison between Pearson’s coefficients in control and 10nM YD treated cells were statistically analysed by unpaired Student’s t-test, ^**#**^*P* < 0.0001.

Because SerpinB2 is an endogenous uPA inhibitor, we next examined the effect of the up-regulation of SerpinB2 by YD on uPA expression. YD increased SerpinB2 protein levels in a time-dependent manner while suppressing uPA (Fig. 5a). YD also increased the mRNA expression of SERPINB2 without affecting UPA mRNA expression (Fig. 5b). This finding suggested that the YD-mediated up-regulation of SerpinB2 may regulate the degradation of the uPA protein and not alter its transcriptional activity. In addition, the mRNA levels of UPA were decreased compared to those of SERPINB2 in the YD-treated cells, and this trend of expression was similar to the MRC5 normal lung cells shown in Fig. 1g. Once the uPAR/uPA-SerpinB2 complex is internalized by its interaction with LRP, the complex is sorted in endosomes for degradation and then degraded in lysosomes^10^,^11^. We observed a significant increase in SerpinB2 expression as shown in Fig. 5a and the co-localization of uPA, SerpinB2 and LAMP1, a lysosomal marker, in H292-Gef cells following a 6-h treatment with 10 nM YD (Fig. 5c,d). In addition, the Pearson’s coefficients between SerpinB2/uPA, SerpinB2/LAMP1 and uPA/LAMP1 were above 0.5, indicating positive for co-localization (Fig. 5e). This result indicated that treatment with YD consequently resulted in the lysosomal degradation of uPA. Taken together, these findings suggest that YD effectively up-regulated SerpinB2 and down-regulated uPA protein expression *in vitro*.

### YD inhibits the MMP2 activity and MAPK signaling, and alters the expression of cadherins in H292-Gef cells

**Figure 6:**
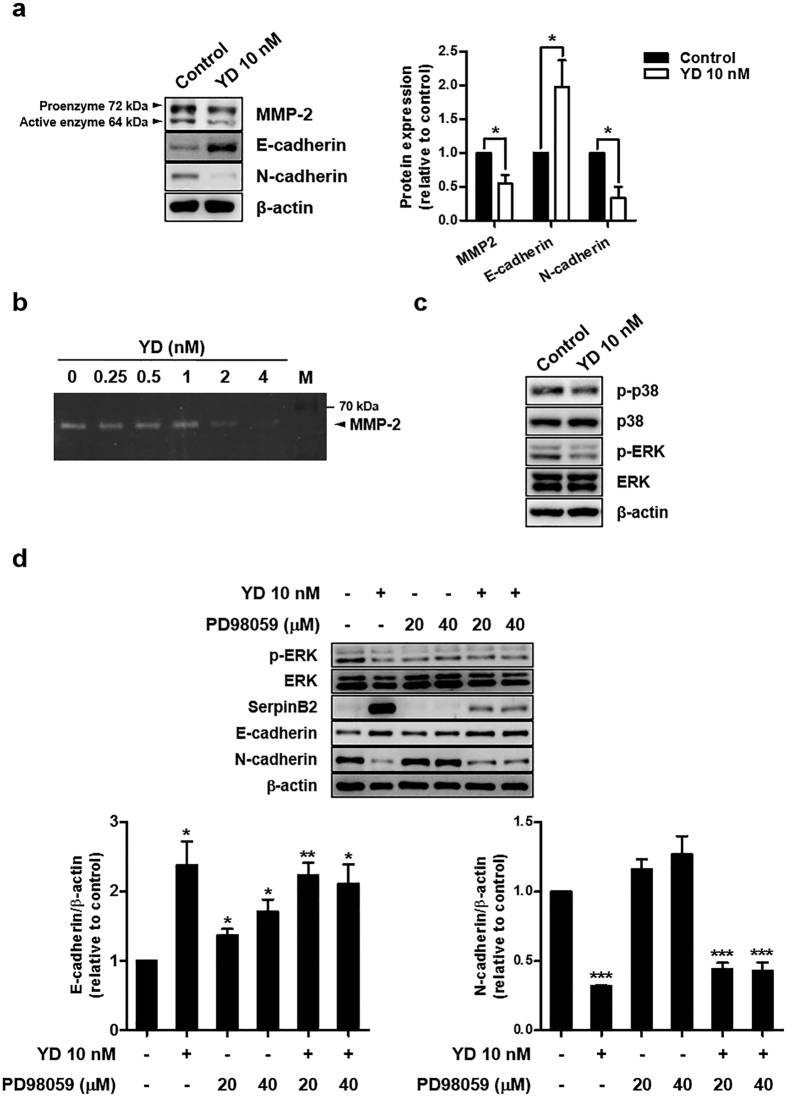
Effects of YD on MMP2, MAPK and cadherins in H292-Gef cells. **(a)** H292-Gef cells were treated with 10 nM YD for 24 h. The protein expression levels of MMP2, E-cadherin and N-cadherin were measured by western blot analysis. The levels of MMP2, E-cadherin and N-cadherin were normalized to β-actin levels, and presented as the mean fold changes ± SD relative to control. **(b)** Gelatin zymography was performed as described in the Methods section. H292-Gef cells were treated with YD for 72 h, and then the proteolytic activity of MMP2 was determined by the degradation of gelatin. M, protein marker. **(c)** Cells were treated with 10 nM YD for 24 h, and cell lysates were subjected to western blot analysis with indicated antibodies. **(d)** Cells co-treated with YD (10 nM) and the MEK inhibitor PD98059 (20, 40 μM) for 24 h were subjected to western blotting for indicated proteins. The levels of E-cadherin and N-cadherin were normalized to β-actin levels, and presented as the mean fold changes ± SD relative to control. β-actin was used as a loading control for western blot analysis, and the protein levels were quantified by densitometry using ImageJ. *P < 0.05, **P < 0.01, ***P < 0.005.

Because uPA is known to activate proMMPs indirectly, the consequential effects of YD on MMPs were further determined in H292-Gef cells. When cells were treated with 10 nM YD for 24 h, MMP-2 expression was suppressed (Fig. 6a). To further examine whether YD inhibits the proteolytic activity of MMP2, gelatin zymography was performed using the conditioned media collected after treatment with YD for 72 h. As shown in Fig. 6b, MMP2 activity was inhibited by YD treatment (2 and 4 nM). The epithelial–mesenchymal transition (EMT) is a process of morphogenesis, in which cells switch from an epithelial phenotype to a mesenchymal phenotype, and this is often observed in metastatic cancers^32^. As the invasiveness of H292-Gef cells was suppressed by YD, we further investigated the levels of EMT markers, such as epithelial E-cadherin and mesenchymal N-cadherin. As shown in Fig. 6a, YD markedly increased E-cadherin expression and decreased N-cadherin expression in H292-Gef cells.

The mitogen activated protein kinase (MAPK) pathway is a well-known pathway that increases cell motility and MMP secretion, leading to the protrusion of invadopodia/podosomes^24^,^33^. Therefore, we also evaluated the effects of YD on the MAPK pathways in H292-Gef cells. The expression of activated p38 and ERK (phosphorylated-p38 (p-p38) and p-ERK) in H292-Gef cells was down-regulated by treatment with 10 nM YD (Fig. 6c). To further understand the relation between YD-mediated ERK and SerpinB2 regulation on EMT, we co-treated H292-Gef cells with YD and PD98059, a MEK1 inhibitor that blocks the MEK1-ERK pathway, and observed changes in E-cadherin and N-cadherin (Fig. 6d). ERK activation was inhibited by YD and PD98059 in H292-Gef cells. Treatment with PD98059 increased the expression of E-cadherin, but did not alter N-cadherin expression. However, the co-treatment of YD and PD98059 increased E-cadherin, but decreased N-cadherin expression. In addition, PD98059 suppressed YD-mediated induction of SerpinB2, suggesting that MEK/ERK pathway might be in part associated with the regulation of SerpinB2 expression. Collectively, the down-regulation of MMP-2, the MAPK pathway, and the modulation of cadherins by YD treatment could be thought to suppress ECM degradation and protrusion of invadopodia-like structures in H292-Gef cells.

**Figure 7:**
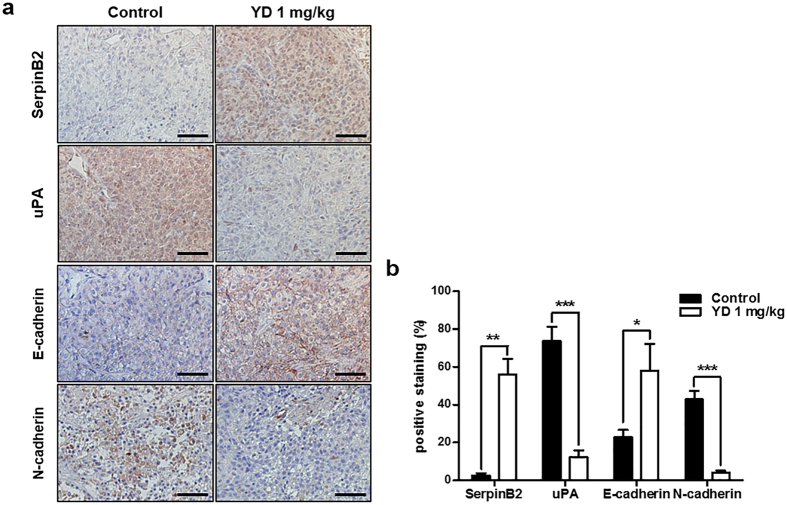
YD-mediated changes in the expression of proteins related to the uPA system, E-cadherin and N-cadherin in xenograft tumors. **(a)** Immunohistochemical analysis of SerpinB2, uPA, E-cadherin and N-cadherin was performed using antibodies against each protein in H292-Gef xenograft tumor sections obtained from a previous study. Scale bar, 50 μm. **(b)** The positive stainings (%) of sections were calculated from the ratio of positive-stained area and total area quantified by ImageJ. The data are presented as the mean ± SD. *P < 0.05, **P < 0.01, ***P < 0.005.

To further validate our *in vitro* findings of the effects of YD on the expression of key markers including SerpinB2, uPA, E-cadherin and N-cadherin, we evaluated the effects of YD in tumor tissues. The tumor sections obtained in our previous *in vivo* study were stained for four markers by immunohistochemistry. YD effectively suppressed tumor growth in H292 and H292-Gef cell-implanted nude mouse xenograft models^28^. Consistent with our *in vitro* findings, the expression of SerpinB2 and E-cadherin was remarkably increased, while uPA and N-cadherin expression were suppressed in tumors of the YD-treated group (1 mg/kg) when compared to the control group (Fig. 7).

## Discussion

The clinical relevance of the expression levels of uPA system components in cancer progression and metastasis has been investigated in various types of cancers^34^. Among these large studies, several reports have suggested that low SerpinB2 expression is a biomarker of metastasis and of poor prognosis for lung cancer^4^,^6^. However, the precise role of SerpinB2 in acquired EGFR-TKI resistance, which is a major barrier in the treatment of lung cancer patients, has not been explored. In this study, the relationship between SerpinB2 and the acquired resistance to gefitinib, an EGFR-TKI, in NSCLC cells was investigated. We first determined that the levels of SerpinB2 were significantly down-regulated in gefitinib-resistant H292 (H292-Gef) cells compared to gefitinib-sensitive H292 cells. This decrease in SerpinB2 expression was also observed in H292 parental cells following gefitinib treatment, while the sensitivity of cells to gefitinib was maintained. These results suggest that the initial down-regulation of SerpinB2 in parental cells does not affect the anti-cancer activity of gefitinib. In addition, these findings may explain that the alteration in SerpinB2 levels observed in acquired gefitinib-resistant cells is resulted from the continuous exposure of cells to gefitinib. SerpinB2 is known to protect retinoblastoma protein (Rb) from degradation, which in turn accumulates in cells during G_0_/G_1_ phase^35^. Therefore, even though gefitinib is known to evoke G_0_/G_1_ phase arrest^36^, the down-regulation of SerpinB2 in H292-Gef cells may lead to cell cycle progression and cell proliferation.

According to clinical statistics, patients with uPA-positive and SerpinB2-negative lung cancers have shown poor survival rates compared to patients positive for both uPA and SerpinB2, and the co-expression of these two markers correlated with the absence of lymph node metastasis^4^,^6^. Furthermore, the significance of SerpinB2 is highly dependent on the expression of uPA in lung cancer and other types including breast, head and neck and oral cancers^14^,^19^,^37^,^38^. In the present study, we observed high uPA and low SerpinB2 expression in NSCLC cells compared to normal lung cells. Moreover, the relative uPA level exceeded that of SerpinB2 in gefitinib-resistant cells, while the opposite was observed in normal cells. These findings may suggest that the loss of balance between SerpinB2 and uPA could induce cancer progression, including metastasis and drug resistance.

The uPA system is known to play a key role in cancer invasion and metastasis through the activation of plasminogen, which in turn induces ECM degradation^39^. In addition, the uPA system is associated with invadopodia protrusion and ECM degradation capacity^25^,^40^. As an inhibitor of uPA, SerpinB2 suppresses the activity of uPA and thus prevents ECM degradation and reduces the length of invadopodia-like structures. Consistent with this role for SerpinB2, H292-Gef cells expressing low SerpinB2 showed increased invasiveness compared to H292 cells. Moreover, the length of the invadopodia-like structures and their protrusion were both increased in H292-Gef cells. Further experiments suppressing or elevating SerpinB2 expression provided additional evidence that SerpinB2 levels are associated with the invasive characteristics observed in cancer cells. However, the effect of SerpinB2 expression on the metastatic capability of gefitinib-sensitive and resistant cells requires further investigation in *in vivo* models.

Cancer cells acquire migratory and invasive properties through actin cytoskeleton remodeling and the formation of invasive structures that facilitate the dissemination of cells^23^. Therefore, based on our results finding that SerpinB2 alters cell invasion and invadopodia-like structure protrusion, we propose the up-regulation of SerpinB2 as a strategy to overcome gefitinib resistance in NSCLC cells. Following the treatment of H292-Gef cells with the antitumor agent YD, the expression of SerpinB2 was increased in a time-dependent manner. Consequently, the migration and invasion of H292-Gef cells were suppressed, and the length of invadopodia-like structures was also decreased. These findings supported the potential of enhancing SerpinB2 to overcome gefitinib resistance in NSCLC cells. Further experiments showed that treatment with YD down-regulated the expression of uPA protein, but not mRNA. The inhibition of active uPAR-bound uPA by SerpinB2 is followed by an internalization of the uPAR/uPA-SerpinB2 complex and lysosomal degradation^10^. Therefore, the co-localization of SerpinB2, uPA and LAMP1 in H292-Gef cells following YD treatment suggests that the YD-induced increase in SerpinB2 expression eventually leads to the lysosomal degradation of uPA. The YD-induced down-regulation of uPA, which indirectly activate proMMPs through activation of plasmin^8^, suppresses the protein expression and enzyme activity of MMP2. This event might influence the ECM degradation, migration and invasion properties of cancer cells.

The MAPK pathways, such as MEK/ERK and p38, are involved in cell proliferation, apoptosis, and motility^41^. Dysregulation of MAPK signaling is known to promote the development and progression of cancer. In this study, YD-induced SerpinB2 expression was decreased by a MEK inhibitor, suggesting that SerpinB2 expression may be in part regulated by MEK/ERK pathway. However, the effect of YD on ERK and SerpinB2 seems to be different because YD up-regulates SerpinB2 expression while ERK is suppressed by YD. Complex of uPA and SerpinE1, another uPA inhibitor, is known to interact with very-low-density lipoprotein receptor (VLDLR) which results in sustained stimulation of ERK phosphorylation and increased cell proliferation^13^,^14^. In contrast, SerpinB2-uPA complex does not directly bind to VLDLR due to lack of a high-affinity-binding site for VLDLR in SerpinB2^42^. Therefore, a plausible mechanism of YD on regulating ERK and SerpinB2 suggests that YD-induced SerpinB2 may compete with SerpinE1 to form SerpinB2-uPA complex, which do not stimulate ERK signaling. Although treatment of YD or MEK inhibitor effectively suppressed ERK activation, co-treatment with YD and MEK inhibitor did not show a synergistic effect on E-cadherin and N-cadherin expression. This finding may be in part related to the suppression of YD-induced SerpinB2 expression by MEK inhibitor. In addition, MAPK4, also known as ERK4, was found to be up-regulated in H292-Gef cells compared to H292 cells by cDNA microarray. Few studies reported that MAPK4 levels are elevated in lung adenomas of oncogenic K-Ras transgenic mice, and silent mutations or single amino acid substitutions in MAPK4 have been detected in lung and skin cancer tissues^43^,^44^. However, the precise biological function of MAPK4 and its association in cancer is still unclear^41^. Thus, the up-regulation of MAPK4 in the acquired gefitinib resistance remains to be further elucidated.

In summary, this study demonstrated the importance of SerpinB2 in the modulation of the invasiveness of acquired-gefitinib-resistant NSCLC cells. Moreover, the induction of SerpinB2 by YD treatment inhibited uPA, invadopodia-like structures and invasive characteristics, including migration, invasion and MMP2 expression. Therefore, these findings support the potential of SerpinB2 as a novel biomarker for acquired-gefitinib resistance and as a therapeutic target for overcoming resistance in human lung cancer patients.

## Methods

### cDNA microarray expression analysis

The gene expression profiles of H292 and H292-Gef cells were determined using Illumina HumanHT-12 v3 Expression BeadChip (Illumina, Inc., San Diego, CA, USA) according to the technical manual of Macrogen (Seoul, Korea). Total RNA was extracted from cells with TRI reagent (Invitrogen, Grand Island, NY, USA) following the manufacturer’s instructions. The intensity and quantity of total RNA was assessed using a Nanodrop ND-1000 spectrometer (Nanodrop Technologies, Wilmington, DE, USA), and 0.55 μg of total RNA was used to prepare biotinylated cRNA using the Illumina TotalPrep RNA Amplification Kit (Ambion, Austin, TX, USA). After fragmentation, 0.75 μg of labeled cRNA were hybridized to the Illumina HumanHT-12 Expression BeadChip following the manufacturer’s protocols. Arrays were scanned with the Illumina Bead Array Reader Confocal Scanner. Array data export processing and analysis was performed using Illumina GenomeStudio v2009.2 (Gene Expression Module v1.5.4).

### Cell culture

Human lung carcinoma H292 cells were obtained from the American Type Culture Collection (Manassas, VA, USA). Embryonic lung fibroblast (MRC-5) cells were provided from the Korean Cell Line Bank (Seoul, Korea). H292 cells were cultured in RPMI 1640 medium, and MRC-5 cells were cultured in MEM medium supplemented with 10% FBS and antibiotics-antimycotics (PSF; 100 units/mL penicillin G sodium, 100 μg/mL streptomycin, and 250 ng/mL amphotericin B). Cells were incubated at 37 °C with 5% CO_2_ in a humidified atmosphere. Gefitinib-resistant H292 cells (H292-Gef) were developed from the parental H292 cells through continuous exposure to gradually increasing concentrations of gefitinib (Selleckchem, Houston, TX, USA) and were maintained in RPMI 1640 medium containing 1 μM gefitinib.

### Western blot analysis

Western blot analysis was performed as described previously^28^, using equal amounts (10 or 50 μg) of protein from each cell lysate. The following antibodies were used: anti-SerpinB2, anti-uPA, anti-LAMP1, anti-phosphorylated p38 (p-p38), anti-p38, anti-phosphorylated ERK (p-ERK), anti-ERK, anti-β-actin (Santa Cruz Biotechnology, Santa Cruz, CA, USA); anti-MMP-2 (Cell Signaling Technology, Danvers, MA, USA); and anti-E-cadherin and anti-N-cadherin (BD Biosciences, San Diego, CA, USA).

### Real-time polymerase chain reaction (PCR)

Total RNA from the cells or tumor tissues was extracted with TRI reagent (Invitrogen, Grand Island, NY, USA), and 1 μg of total RNA was reverse-transcribed using a Reverse Transcription System (Promega, Madison, MA, USA) according to the manufacturer’s instructions. Real-time PCR was conducted using iQ^TM^ SYBR^®^ Green Supermix (Bio-Rad, Hercules, CA, USA) according to the manufacturer’s instructions. The conditions for the assay were as follows: 20 sec at 95 °C; 40 cycles of 20 sec at 95 °C, 20 sec at 56 °C, and 30 sec at 72 °C; 1 min at 95 °C; and 1 min at 55 °C. All of the experiments were performed in triplicate, and the analysis was performed using the comparative C_T_ method using β-actin for normalization. Gene-specific primers for real-time PCR were synthesized from Bioneer (Daejeon, Korea), and the sequences of the primers are listed in Supplementary Table S1.

### Immunohistochemistry of tumors

Xenograft tumors were obtained from our previous *in vivo* study^28^. The excised tumors were fixed in 4% paraformaldehyde (in PBS) and embedded in paraffin. Sectioned slides of the embedded specimens were serially deparaffinized, rehydrated, and subjected to antigen retrieval. The slides were incubated with anti-SerpinB2, anti-uPA, anti-E-cadherin or anti-N-cadherin antibodies and detected using the LSAB^TM^+ System-HRP kit (Dako, Glostrup, Denmark) followed by counterstaining with hematoxylin. The stained sections were observed and photographed with an inverted phase-contrast microscope.

### Invasion assay

The invasion assay was performed using 24-well Transwell inserts with 6.5-mm diameters and 8.0-μm pore-size membranes (Corning Incorporated, Corning, NY, USA). The upper surface of the membrane was pre-coated with Matrigel (BD Biosciences), and the lower surface was coated with gelatin (Sigma Aldrich, St. Louis, MO, USA). The lower chambers contained medium supplemented with 10% FBS. Cells (2 × 10^5 ^cells/100 μl) were suspended in serum-free medium with or without compound and then seeded into the upper chambers. Following a 24- or 48-h incubation, the non-invaded cells in the upper chamber were removed, and the invaded cells on the lower surface of the membrane were fixed with 4% paraformaldehyde (in PBS). The fixed cells were stained with 1% crystal violet and were then counted and visualized under an inverted phase contrast light microscope.

### Cell culture in 3-dimensional (3D) type I collagen gels

3D cell culture in type I collagen was performed as described previously^45^ with modifications. Briefly, collagen I mixtures were prepared by adding 10× PBS, ice-cold 1 N NaOH and media to rat tail type I collagen (BD Biosciences) to a final concentration of 2.5 mg/ml and pH 7.2–7.4. The mixtures were centrifuged (3 min, 10,000 × g, 4 °C) to eliminate air bubbles and placed on ice until use. A cover slip in a culture dish was first covered with 150 μl of collagen solution for the bottom layer, which was allowed to gel at 37 °C for 30 min. A cell suspension containing 1 × 10^5 ^cells/100 μl was mixed with 100 μl of collagen solution, loaded onto the bottom layer and allowed to gel at 37 °C for 30 min. Culture medium was then added to the dish, and the cells were incubated for 2 days prior to compound treatment. The embedded cells were observed under an inverted phase contrast light microscope or subjected to immunocytochemistry.

### Immunocytochemistry

Cells were grown on cover slips in dishes. Following treatment, the cells were fixed with 4% paraformaldehyde (in PBS) for 15 min. The fixed cells were blocked with 1% BSA (in PBS) containing 0.1% Triton X-100 for 30 min at room temperature. The cells were incubated with the indicated primary antibodies at 4 °C overnight. Alexa Fluor^®^ 568 Phalloidin was obtained from Invitrogen and anti-Tks5 was purchased from Santa Cruz Biotechnology. Following an overnight incubation, the cells were incubated with the corresponding fluorescent-conjugated secondary antibodies for 2 h at room temperature. DAPI (0.5 μg/ml; Vector Laboratories, Burlingame, CA, USA) was used to counterstain the nuclei. The images were obtained using a Leica TCP-SP8 confocal microscope (Leica, Wetzlar, Germany) and Zeiss ApoTome microscope (Carl Zeiss, Jena, Germany). The co-localization rate and Pearson’s co-localization coefficients were determined with LAS AF Image software (Leica). Co-localization rate is the value that indicates the extent of co-localization in percentage and is calculated as follows: 100 × (Co-localization Area/Area Foreground), where area foreground is the difference between total area of the image and area of the image background. The values of Pearson’s coefficients ranging from 0.5 to 1 are considered positive co-localization.

### Plasmid construction and transfection

The plasmid expressing full-length SerpinB2 cDNA was purchased from the Korea Human Gene Bank (Daejeon, Korea) and was sequence verified to be 100% identical to the published sequence, NM_002575. The SerpinB2 cDNA was amplified with a pair of primers (5′-GCGCTCGAGATGGAGGATCTTTGTGTGGCAAACACAC-3′ and 5′-CGCGAATTCTGGGTGAGGAAAATCTGCCGAAAAATAAAATG-3′) by PCR. The amplified SerpinB2 cDNA was inserted between the *Xho*I and *Eco*RI restriction sites of pEGFP-N1 and was sequence verified. For transient transfection, cells were transfected with 0.5–6 μg pEGFP-N1 or pEGFP-N1-SERPINB2 using FuGENE^®^ HD (Promega) according to the manufacturer’s instructions.

### RNA interference

RNA interference of SERPINB2 was performed using 25-bp siRNA duplexes purchased from Invitrogen. The coding strand for SERPINB2 was as follows: sense 5′-AAAUUGGCCCGUCCUUGUUGAAGG-3′ and antisense 5′-CCUUCAACAAGGGACGGGCCAAUUU-3′. Cells were transfected with 10 nM siRNA duplexes using Lipofectamine RNAiMAX (Invitrogen) according to the manufacturer’s instructions. Cells transfected with a control nonspecific siRNA duplex (Invitrogen) were used as controls for direct comparison.

### Cell viability assay

Cell viability was measured using the MTT assay. Cells were seeded in 96-well plates at 2 × 10^4^ cells per well. After a 24-h incubation period, cells were treated with various concentrations of YD. At 24 or 48 h, MTT (5 mg/ml in PBS) was added to the cells at a final concentration of 0.5 mg/ml and incubated for an additional 4 h. The resulting formazan crystals were dissolved in DMSO, and the absorbance was read at 595 nm using a VersaMax ELISA microplate reader (Molecular Devices, Sunnyvale, CA, USA). Cell viability was calculated as a percentage relative to vehicle-treated control. YD (purity > 98.5%) was isolated and identified from a CHCl_3_-soluble fraction of the flower buds of *Daphne genkwa*, as described previously^27^,^31^.

### Wound-healing assay

Cells were grown to 80–90% confluence in a 12-well plate. A wound was created by mechanically scratching the monolayer of cells with a 200-μl pipette tip. The cultures were washed with PBS to remove the detached cells and then incubated with serum-free medium containing various concentrations of compound for 30 h. Images of the wounds were taken at 0 and 30 h under an inverted phase contrast light microscope. The percentage of cell migration was calculated as follows: cell migration (%) = 100 × [(A_0h_ − A_30h_)_compound_/(A_0h_ − A_30h_)_control_], where A is the average of cell free area quantified by ImageJ programme (1.44p, National Institutes of Health, USA).

### Gelatin Zymography

Cells were treated with various concentrations of YD using serum-free medium. After 72 h of incubation, conditioned media were collected and subjected to gelatin zymography for determination of MMP-2 expression and activity. Briefly, the culture supernatants were re-suspended in 5× non-reducing loading buffer and electrophoresed on a polyacrylamide gel containing 0.1% gelatin. After electrophoresis, the gel was washed twice with a buffer containing 50 mM Tris–HCl (pH 7.5), 100 mM NaCl and 2.5% Triton X-100 to remove SDS. Subsequently, the gel was incubated in a developing buffer (50 mM Tris-HCl, pH 7.5, 150 mM NaCl, 10 mM CaCl_2_, 0.02% NaN_3_, and 1 μM ZnCl_2_) for 24 h at 37 °C. The gel was then stained with Coomassie Brilliant Blue R-250, and de-stained with 5% methanol and 7% acetic acid until clear bands indicating proteolytic activities were revealed.

### Statistical analysis

The data are presented as the mean ± SD for the indicated number of independently performed experiments. The statistical significance (*P* < 0.05) was assessed using Student’s t-test. All statistical tests were two-sided.

## Additional Information

**How to cite this article**: Bae, S. Y. *et al*. Down-regulation of SerpinB2 is associated with gefitinib resistance in non-small cell lung cancer and enhances invadopodia-like structure protrusions. *Sci. Rep*. **6**, 32258; doi: 10.1038/srep32258 (2016).

## Supplementary Material

Supplementary Information

## Figures and Tables

**Table 1:** List of the top 11 probes and 10 genes up-regulated (a) and 13 probes and 10 genes down-regulated (b) in H292-Gef cells compared to H292 cells.

Probe ID	Gene symbol	Fold change	Description	Accession
(a) H292-Gef versus H292 up-regulated				
ILMN_1795325	ACTG2	19.58	Actin, gamma 2, smooth muscle, enteric	NM_001615
ILMN_1801216	S100P	8.62	S100 calcium binding protein P	NM_005980
ILMN_1729801	S100A8	6.80	S100 calcium binding protein A8	NM_002964
ILMN_2093343	PLAC8	6.13	Placenta-specific 8	NM_016619
ILMN_2112128	MAPK4	5.83	Mitogen-activated protein kinase 4	NM_002747
ILMN_1653026	PLAC8	5.15	Placenta-specific 8	NM_016619
ILMN_2149164	SFRP1	4.54	Secreted frizzled-related protein 1	NM_003012
ILMN_1689329	SCD	4.37	Stearoyl-CoA desaturase (delta-9-desaturase)	NM_005063
ILMN_1715508	NNMT	4.33	Nicotinamide N-methyltransferase	NM_006169
ILMN_1750974	S100A9	3.87	S100 calcium binding protein A9 (calgranulin B)	NM_002965
ILMN_1723035	OLR1	3.79	Oxidized low density lipoprotein (lectin-like) receptor 1	NM_002543
(b) H292-Gef versus H292 down-regulated				
ILMN_2150851	SERPINB2	−21.64	Serpin peptidase inhibitor, clade B (ovalbumin), member 2	NM_002575
ILMN_2212878	ESM1	−6.97	Endothelial cell-specific molecule 1	NM_007036
ILMN_1688892	LAMA3	−6.45	Laminin, alpha 3	NM_198129
ILMN_2406035	LAMA3	−6.16	Laminin, alpha 3	NM_198129
ILMN_2219002	KRT6A	−5.62	Keratin 6A, type II	NM_005554
ILMN_2150856	SERPINB2	−5.57	Serpin peptidase inhibitor, clade B (ovalbumin), member 2	NM_002575
ILMN_1651365	ZBED2	−5.45	Zinc finger, BED-type containing 2	NM_024508
ILMN_1707727	ANGPTL4	−4.82	Angiopoietin-like 4	NM_139314
ILMN_1653824	LAMC2	−4.32	Laminin, gamma 2	NM_005562
ILMN_1701424	LAMC2	−4.17	Laminin, gamma 2	NM_005562
ILMN_1798181	IRF7	−4.15	Interferon regulatory factor 7	NM_004029
ILMN_1695590	ADRB2	−4.08	Adrenoceptor beta 2, surface	NM_000024
ILMN_1674620	SGCE	−3.98	Sarcoglycan, epsilon	NM_001099400
