# Supplementary material for: Down-regulation of SerpinB2 is associated with gefitinib resistance in non-small cell lung cancer and enhances invadopodia-like structure protrusions
Source: Sci Rep. 2016 Aug 25;6:32258. doi: 10.1038/srep32258 (PMC4997607; doi:10.1038/srep32258)
Supplement: Supplementary Information [file srep32258-s1.pdf]

Scientific Reports

Down-regulation of SerpinB2 is associated with gefitinib resistance in non-small cell lung cancer and enhances invadopodia-like structure protrusions

Song Yi Bae, Hyen Joo Park, Ji-Young Hong, Hye-Jung Lee, Sang Kook Lee*

College of Pharmacy, Natural Products Research Institute, Seoul National University, Seoul 151-742, Korea

***Corresponding Author:**

Sang Kook Lee

College of Pharmacy, Seoul National University

1 Gwanak-ro, Gwanak-gu, Seoul 151-742, Korea

Phone: +82-2-880-2475; Fax: +82-2-762-8322; E-mail: sklee61@snu.ac.kr

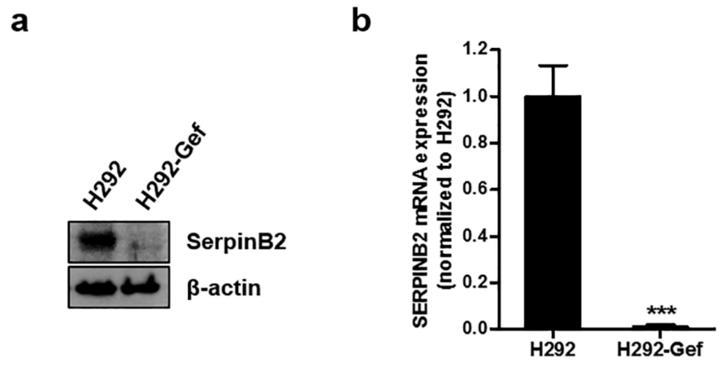

Supplementary Figure S1. Down-regulated SerpinB2 expression in H292-Gef xenograft tumors compared to H292 xenograft tumors.

(a,b) SerpinB2 protein (a) and mRNA (b) expressions in the frozen xenograft tumor tissue samples were investigated by western blot and real-time PCR using β-actin as the internal control. The mRNA levels are presented as the mean fold changes ± SD relative to the H292 control.*$P < 0.05$, **$P < 0.01$, ***$P < 0.005$.

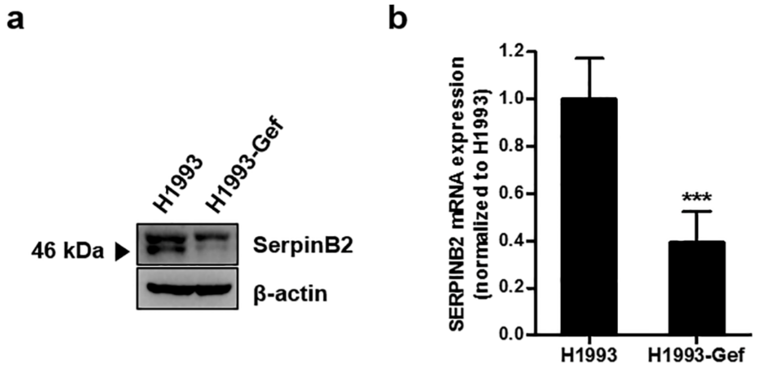

Supplementary Figure S2. Down-regulated SerpinB2 expression in H1993-Gef cells, an acquired gefitinib-resistant non-small cell lung cancer cell line.

(a,b) SerpinB2 protein (a) and mRNA (b) expressions in H1993 and H1993-Gef cells were investigated by western blot and real-time PCR using β-actin as the internal control. The mRNA levels are presented as the mean fold changes ± SD relative to the H1993 control. *P < 0.05, **P < 0.01, ***P < 0.005.

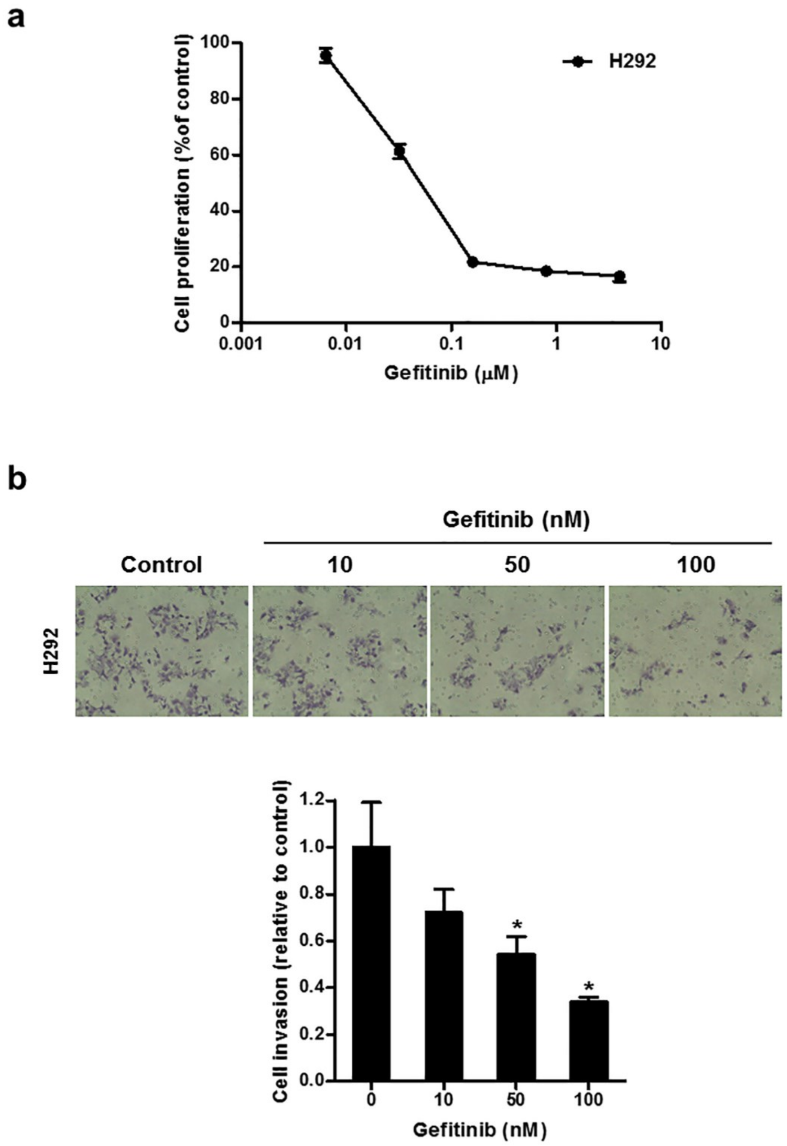

Supplementary Figure S3. Effect of gefitinib on cell proliferation and invasion in H292 cells.

(a) H292 cells were treated with gefitinib for 72 h, and cell proliferation was measured by SRB assay. The data are presented as the mean ± SD. **(b)** H292 cells were added to Matrigel-coated transwell inserts with gefitinib and incubated for 24 h. The invaded cells were fixed, stained and counted as described in the Methods section. Magnification, x 100. The data are presented as the mean ± SD. **P* < 0.05, ***P* < 0.01, ****P* < 0.005.

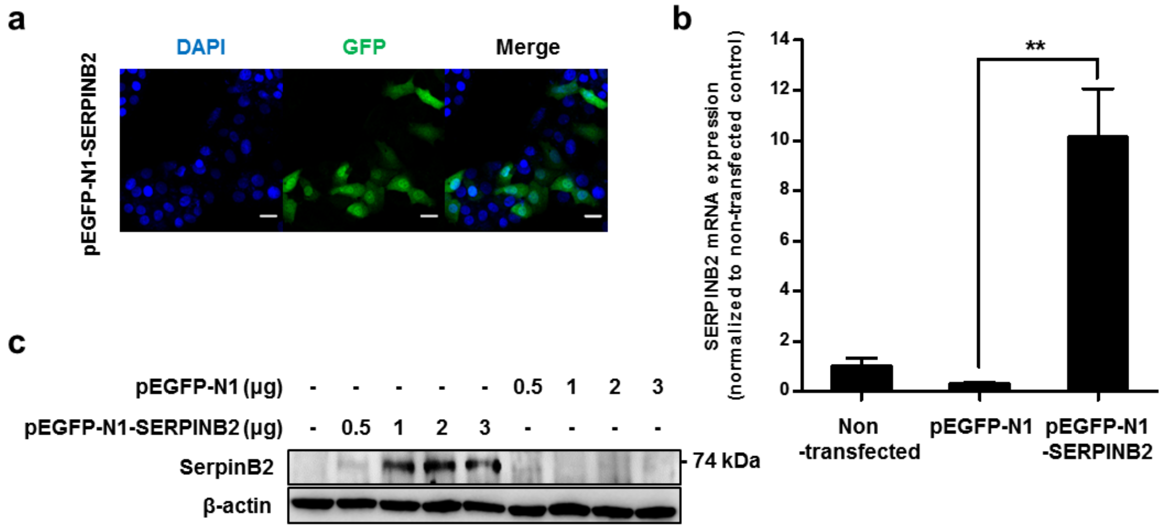

Supplementary Figure S4. Evaluation of the constructed plasmid, pEGFP-N1-SERPINB2, for SerpinB2 overexpression in H292-Gef cells.

H292-Gef cells were transfected with the indicated plasmids for 24 h. **(a)** pEGFP-N1-SERPINB2-transfected cells were fixed and the nuclei were stained with DAPI. Images were taken by confocal laser microscope, and GFP fluorescence indicates the transfected cells. Scale bars, 20 μm. **(b)** The mRNA levels of SERPINB2 were examined, and β-actin was used for normalization. The data are presented as the mean fold changes ± SD relative to the non-transfected control. **(c)** Protein levels of SerpinB2 were detected with anti-SerpinB2 using β-actin as a loading control. *$P < 0.05$, **$P < 0.01$, ***$P < 0.005$.

Supplementary Table S1. Sequences of target gene-specific primers used for real-time PCR.

**Target genes**		**Sequences**
SERPINB2	Sense	5′-CAT GGA GCA TCT CGT CCA C-3′
	Antisense	5′-ACT GCA TTG GCT CCC ACT T-3′
UPA (PLAU)	Sense	5′-GTA CAA CTC CCG GCA CGA-3′
	Antisense	5′-TGA CTG GCA GGA ACT CCA C-3′
β-Actin	Sense	5’-AGC ACA ATG AAG ATC AAG AT-3’
	Antisense	5’-TGT AAC GCA ACT AAG TCA TA-3’

Supplementary Methods

Ex vivo biochemical analysis of tumors

A portion of the frozen tumors was thawed on ice and homogenized using a hand-held homogenizer in Complete Lysis Buffer (Active Motif, Carlsbad, CA, USA). The protein concentrations of tumor lysates were determined, and then samples were aliquoted and stored at -80°C. Prior to western blot analysis, the lysates were boiled with 2× Laemmli sample buffer at 95°C for 10 min.

Cell culture

Human lung carcinoma H1993 cells were purchased from the American Type Culture Collection (Manassas, VA, USA). H1993 cells were grown in RPMI 1640 medium supplemented with 10% FBS and antibiotics-antimycotics (PSF; 100 units/mL penicillin G sodium, 100 μg/mL streptomycin, and 250 ng/mL amphotericin B). Cells were incubated at 37°C with 5% CO_2 in a humidified atmosphere. Gefitinib-resistant H1993 cells (H1993-Gef) were developed from the parental H1993 cells through continuous exposure to gradually increasing concentrations of gefitinib (Selleckchem, Houston, TX, USA) and were maintained in RPMI 1640 medium containing 10 μM gefitinib.

Cell Proliferation Assay

Cells (2×10^4 cells per well) were treated with various concentrations of gefitinib in 96-well plates. After 72h incubation, cells were fixed with 10% TCA solution, and the cell proliferation was determined through a sulforhodamine B (SRB) assay. The percentage of cell proliferation was calculated as follows: cell proliferation (%) = $100 \times [(A_{\text{treated}} - A_{\text{zero day}})/(A_{\text{control}} - A_{\text{zero day}})]$, where A is the average absorbance. The IC_{50} values were determined by non-linear regression analysis using TableCurve 2D v5.01 (Systat Software Inc., San Jose, CA, USA).
